# Gender Differences in Objective and Subjective Social Isolation and Self-Reported Hypertension in Older Adults

**DOI:** 10.3390/jcdd12040136

**Published:** 2025-04-04

**Authors:** Tyrone C. Hamler, Ann W. Nguyen, Harry Owen Taylor, Weidi Qin, Fei Wang

**Affiliations:** 1 Graduate School of Social Work, University of Denver, Denver, CO 80210, USA; 2 Jack, Joseph and Morton Mandel School of Applied Social Sciences, Case Western Reserve University, Cleveland, OH 44106, USA; nguyena@case.edu; 3 Factor-Inwentash Faculty of Social Work, University of Toronto, Toronto, ON M5S 1V4, Canada; harry.taylor@utoronto.ca; 4 Sandra Rosenbaum School of Social Work, University of Wisconsin-Madison, Madison, WI 53706, USA; wqin8@wisc.edu; 5 College of Social Work, University of Tennessee-Knoxville, Knoxville, TN 37210, USA; fwang27@utk.edu

**Keywords:** social support, loneliness, health outcomes, social isolation

## Abstract

Hypertension is a major public health concern, especially in older adults, and gender differences are a factor in terms of its prevalence. Social connections benefit health, while social isolation is linked to negative outcomes. Prior studies suggest social isolation and connectedness vary by gender, but few have explored this relationship with hypertension. This study examined gender differences in the association between social isolation and hypertension in older adults using data from the National Survey of American Life (1280 adults aged ≥55). Weighted logistic regressions tested gender differences in objective and subjective social isolation and hypertension. Both men and women who were objectively isolated from family and friends, or only friends, were less likely to have hypertension than those not isolated. However, when accounting for subjective isolation, only isolation from family predicted hypertension. Gender moderated this relationship—men isolated from family and friends had a higher likelihood of hypertension, while no such association was found for women. Findings suggest that preventing objective isolation, particularly from family, may help reduce hypertension risk in older adults. This study highlights the need to further investigate social isolation’s impact on health and its underlying mechanisms among older adults in the U.S.

## 1. Introduction

Hypertension is a significant public health problem that affects approximately 116.4 million Americans, or 46% of adults in the United States, and is the primary modifiable risk factor for cardiovascular events [1]. In addition to being a highly prevalent health condition, hypertension is also costly, totaling USD 55.9 billion annually in direct and indirect costs [2]. Hypertension, also known as high blood pressure, is more common in older adulthood compared to younger adulthood. The National Institute on Aging (NIA) identifies age as one of the risk factors for hypertension and reports that “as you get older, high blood pressure, especially isolated systolic hypertension, is more common and can increase your risk of serious health problems” [3]. Studies have documented the increase in hypertension prevalence with advanced age [4,5]. Accordingly, the prevalence of hypertension is greatest among older adults, with recent evidence indicating that 70% of older adults have hypertension, compared to 32% of adults aged 40–59 years [6]; therefore, hypertension is a particularly important public health problem for older adults. Gender has also been noted to have a complex relationship with hypertension prevalence and age. Men have a greater chance of having high blood pressure before age 55, while women are more likely to develop high blood pressure after menopause [3].

Researchers have consistently found that having reliable social connections, social resources, and social support networks confers many benefits to health and well-being [7]. The social networks of older adults (in comparison to younger adults) tend to be smaller, possibly due to life events, including retirement, relocation, or the loss of family members, friends, and neighbors [8]. Therefore, older adults may experience greater social isolation compared to younger adults. Those who have access to high-quality connections can confide their worries to others and seek social support from their support networks [9]. In contrast, social isolation is associated with many negative health outcomes, including hypertension [10], mortality [11,12,13,14], worse self-rated health [15,16], emphysema, diabetes, stroke, arthritis, and heart disease [10]. In fact, previous studies have found the effects of social isolation on mortality risk are equivalent to smoking 15 cigarettes per day [17]. Moreover, greater social isolation among older adults is associated with increased Medicare spending [18]; on average annually, socially isolated older adults had USD 1644 greater healthcare expenditures compared to older adults who were not socially isolated. The issue of social isolation has received considerable attention in the media and among prominent national and international organizations, including the Office of the Surgeon General [19], the Institute of Medicine [20], the AARP Foundation [21], the American Public Health Association [22], and the World Health Organization [23]. Notably, the U.S. Surgeon General [19] recently declared social isolation a public health epidemic. Yet, few studies have explored how the social isolation-health connection may vary between men and women. Understanding these differences is particularly important, as research has demonstrated that men’s and women’s relationships differ in several ways, and the association between social relationships and health can vary by gender [24]. Thus, this study aims to determine whether the association between social isolation and hypertension among older adults varies by gender.

### 1.1. Objective and Subjective Social Isolation

Social isolation can be differentiated into two different dimensions: objective and subjective isolation [15,21]. Objective social isolation is defined as a paucity of social contact with members of one’s social network [25]. Subjective social isolation, however, is defined as not feeling particularly close to members of one’s social network, including family members and friends [26,27]. It is important to also note these conditions are not mutually exclusive; an individual can be (1) neither objectively nor subjectively socially isolated, (2) lacking contact with family members and friends but still feel close to them (objectively isolated but not subjectively isolated), (3) have frequent interactions with family and friends but feel emotionally distant (subjectively isolated but not objectively isolated), or (4) both objectively and subjectively isolated [26,27].

The prevalence rate of objective social isolation is estimated at approximately 15–40% of older adults [19]. This range is large because objective isolation is operationalized in numerous ways, and different samples were used to examine objective isolation. Chatters and colleagues found that 24% of older adults are objectively isolated from either family members, friends, or both groups [24]. Regarding subjective social isolation, Chatters and colleagues also noted that approximately 15% of all older adults have some degree of subjective isolation (being subjectively isolated from family only, friends only, or both) [24].

### 1.2. Social Isolation and Hypertension

Previous studies examining social isolation have found mixed results. Udell and colleagues reported that those who lived alone were significantly more likely to have hypertension compared to those with other living arrangements [28]. Nevertheless, there is also research that found no association between social isolation and hypertension [29]. This evidence suggests that living alone, which is only one potential indicator of social isolation, may influence the likelihood of having hypertension. Moreover, these findings warrant further investigation to delineate the connection between social isolation and hypertension. A more nuanced understanding of the effects of social isolation on hypertension would help identify specific aspects of social isolation that are risk factors of hypertension and inform interventions targeting social isolation.

### 1.3. Social Isolation, Social Relationships, and Gender

There are noted gender differences in social isolation and in health outcomes associated with social isolation [24]. Overall, men are consistently more objectively isolated than women [13,24,26]. Generally, it has also been found that throughout the life course, boys/men are more isolated than girls/women, with the gender difference becoming more pronounced for individuals who never marry [30].

Considering differences in social relationships among men and women provides an important context for understanding gender differences in social isolation. Men are less likely to contact family members [24,26], to have large social networks [31], and to participate in social activities, such as religious services or volunteer work, in comparison to women [32]. Women also tend to take on more kin-related communications (i.e., kinkeeping) and are more intensely invested at a psychological level in their relationships while still having social responsibilities to perform outside of the home [33]. Women may also be sensitive to the quality of their social interactions, particularly negative ones, which may have a more potent impact on psychological well-being than positive interactions [34,35].

### 1.4. Focus of the Present Investigation

It is understood that individuals are more likely to be diagnosed with hypertension as they age and that prior research has found mixed findings regarding the relationship between social isolation and hypertension. Gender differences in the relationship between social isolation and hypertension may contribute to the equivocal findings in this area. Despite the extant research on isolation and hypertension among older adults, it remains unclear as to the gender difference in the isolation–hypertension association, with little research focusing on gender-specific differences in this association. In society, men and women are subject to different social expectations, occupy different social roles and have distinct responsibilities within their families and social networks. The disparity in these roles continues into older age. It stands to reason that women may have more opportunities for social contact, but it is not known if these contacts translate into lower levels of objective or subjective social isolation and if this influences hypertension.

Currently, our limited knowledge of gender differences in social isolation limits our understanding of the development of social isolation assessments, services, and interventions for older adults. To address the knowledge gaps in the health effects of social isolation among older adults and associated gender differences, this study aims to determine (1) whether objective and subjective social isolation are associated with hypertension and (2) whether these associations vary by gender in a nationally representative sample of older adults.

## 2. Materials and Methods

### 2.1. Sample

The National Survey of American Life: Coping with Stress in the 21st Century (NSAL) is a nationally representative survey and was collected by the Program for Research on Black Americans at the University of Michigan’s Institute for Social Research. The African American sample is the core sample of the NSAL. The fieldwork for the study was completed by the Institute for Social Research’s Survey Research Center in cooperation with the Program for Research on Black Americans. The NSAL sample utilized a national multi-stage probability design consisting of 64 primary sampling units (PSUs). A total of 6082 face-to-face interviews were conducted with persons aged 18 or older, including 3570 African Americans, 891 non-Hispanic whites, and 1621 Black people of Caribbean descent (see [36] for a more detailed discussion of the NSAL sample). The Caribbean Black sample was selected from two area probability sampling frames: the core NSAL sample and an area probability sample of housing units from geographic areas with a relatively high density of persons of Caribbean descent. Respondents were considered Caribbean Black if they indicated that they were Black and answered affirmatively when asked if they were of West Indian or Caribbean descent, said they were from a country included on a list of Caribbean area countries presented by the interviewers, or stated that their parents or grandparents were born in a Caribbean country. After listwise deletion of cases due to missing data, the analytic sample includes 1278 respondents aged 55 and older.

### 2.2. Measures

#### 2.2.1. Hypertension

Hypertension was assessed using a self-report measure. Respondents were asked to report the presence of hypertension that has been diagnosed by a doctor or health professional by responding yes or no.

#### 2.2.2. Objective Social Isolation

Objective social isolation was operationalized by combining the frequency of contact with family members and the frequency of contact with friends. Frequency of contact with family was assessed by the following item: “How often do you see, write or talk on the telephone with family or relatives who do not live with you? Would you say nearly every day, at least once a week, a few times a month, at least once a month, a few times a year, hardly ever or never?” The frequency of contact with friends was assessed in the same manner as the frequency of contact with family. Both frequency of contact items were recoded into two separate dichotomous variables—objective isolation from family and objective isolation from friends—by combining the following response categories: not objectively isolated (nearly every day, at least once a week, a few times a month) versus objectively isolated (at least once a month, a few times a year, hardly ever or never). The two dichotomous variables, objective social isolation from family and objective social isolation from friends, were then combined into a single variable. This resulted in a four-category, nominal objective isolation variable: (a) objectively isolated from family members and friends, (b) objectively isolated from family, (c) objectively isolated from friends, and (d) not objectively isolated from either group (i.e., family and friends). This operationalization has the advantage of assessing interactions involving both family and friends and provides a more nuanced perspective on social isolation [26,37].

#### 2.2.3. Subjective Social Isolation

A similar coding strategy was used to create subjective social isolation. Subjective social isolation was operationalized by combining both subjective family closeness and subjective friend closeness. Subjective family closeness is assessed by the item, “How close do you feel towards your family members? Would you say very close, fairly close, not too close, or not close at all?” Subjective friend closeness was assessed in the same manner as subjective family closeness. Both subjective closeness items were recoded into two separate dichotomous variables—subjective isolation from family and subjective isolation from friends—by combining the following response categories: not subjectively isolated (very close and fairly close) versus subjectively isolated (not too close and not close at all). These two dichotomous variables were then combined to create a single four-category nominal variable representing respondents who are (a) subjectively isolated from family and friends, (b) subjectively isolated from family only, (c) subjectively isolated from friends only, and (d) not subjectively isolated from either group.

#### 2.2.4. Gender and Covariates

Gender (moderator) was measured dichotomously (male and female). Covariates included race, age, educational attainment, family income, marital status, region, physical activity, body mass index (BMI), and 12-month major depressive disorder (MDD). Age, education, and family income were measured continuously; age and education were assessed in years. Family income was coded in United States dollars. Due to its skewed distribution, we log-transformed family income for multivariate analyses. Missing data for family income and education were imputed using an iterative regression-based multiple imputation approach incorporating information about age, gender, region, race, employment status, marital status, home ownership, and nativity of household residents [38]. Marital status was coded to differentiate respondents who were (1) married or cohabiting and (2) separated, divorced, widowed, or never married. The region was coded dichotomously to those living in the South and those living in other U.S. regions. Physical activity was assessed using a three-item index. These items asked, “How often do you: (a) work in the garden or the yard; (b) engage in active sports or exercise; (c) take walks? Would you say often (4), sometimes (3), rarely (2), or never (1)?” A mean physical activity score was derived from these three items. BMI was a continuous measure calculated as BMI = 703 × weight (lbs.)/height (ins.)^2^. MDD was assessed using the DSM-IV World Mental Health Composite International Diagnostic Interview (WMH-CIDI). The WMH-CIDI is a fully structured diagnostic interview [39]).

### 2.3. Analysis Strategy

We used multivariable logistic regression to test the association between objective and subjective social isolation and hypertension and gender differences in these associations. We constructed interaction terms between (1) objective isolation and gender and (2) subjective isolation and gender to test whether the associations between objective and subjective isolation and hypertension varied by gender. These interaction terms were individually tested in separate multivariable logistic regression models. The significant interaction was depicted using predicted probabilities of hypertension. We also conducted a simple effects analysis to probe this significant interaction. Multivariable logistic regression analyses controlled for sociodemographic and health covariates. All analyses were conducted using Stata 15 [40], which uses the Taylor expansion approximation technique for calculating complex design-based estimates of variance. All statistical analyses accounted for the complex multistage-clustered design of the NSAL sample, unequal probabilities of selection, nonresponse, and post-stratification to calculate weighted, nationally representative population estimates and standard errors.

## 3. Results

Table 1 presents the descriptive statistics for the sample. Slightly over half of the sample (55%) was women, and the mean age was 67 years. On average, respondents had 11.7 years of formal education. The mean family income was USD 30,942. Close to half (46.02%) of all respondents were either married or cohabiting, and slightly over half of the sample (56%) resided in the South. Approximately 5% of respondents reported being objectively isolated from both family and friends, and even fewer respondents (1%) reported being subjectively isolated from both groups. In general, objective isolation rates were higher than subjective isolation rates. Most respondents were neither subjectively (86%) nor objectively (78%) isolated from family and friends. A greater proportion of respondents were subjectively and objectively isolated from friends only as compared to subjective and objective isolation from family only and both family and friends. More than half of all respondents reported being diagnosed with hypertension. A total of 57% of men and 60% of women reported a hypertension diagnosis. Bivariate analysis indicated no statistically significant difference in the prevalence of hypertension between men and women. No data sparseness was detected, as crosstabs among categorical variables showed that all expected cell sizes ≥10 [41].

Table 2 presents the results from the multivariable weighted logistic regressions for hypertension. Model 1 tested the association between objective isolation and hypertension. Objective isolation from (1) family and friends and (2) from family only were both negatively associated with hypertension. That is, compared to the respondents who were not objectively isolated from either group, the respondents who were objectively isolated from both family and friends or family only were less likely to report hypertension. In Model 2, subjective isolation was added to the model. With subjective isolation accounted for, objective isolation from both family and friends attenuated and was no longer significant. Nevertheless, the negative association between objective isolation from family only remained significant. None of the subjective isolation categories were associated with hypertension in this model.

**Figure 1:**
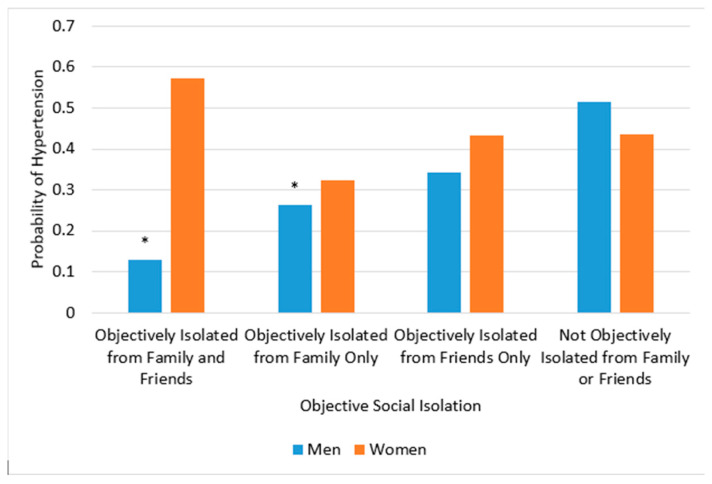
Objective Social Isolation by Women Interaction Effects. Objective isolation and Hypertension Among Men and Women; * denotes significant simple effects at the *p* < 0.05 level. The reference category for this figure is not objectively isolated from family or friends.

In Models 3 and 4, the interaction terms “objective isolation*gender” and “subjective isolation*gender” were added to test gender differences in the associations between objective and subjective isolation and hypertension. We found a significant interaction between gender and objective isolation in Model 3, meaning the association between objective isolation and hypertension varied by gender (Figure 1). More specifically, the interaction category of objective isolation from both family and friends × women was significant. The simple effects analysis indicated that men who were objectively isolated from both family and friends were significantly less likely to have a hypertension diagnosis compared to men who were not objectively isolated from either group (*B* = −0.35, *SE* = 0.10, *t* = −3.47, *p* < 0.01). The interaction category of objective isolation from family only × women was not significant, but the simple effects analysis was significant. Specifically, men who were objectively isolated from family only were significantly less likely to have a hypertension diagnosis compared to men who were not objectively isolated from either family or friends (*B* = −0.22, *SE* = 0.07, *t* = −2.95, *p* < 0.01). In contrast, the simple effects analysis revealed that women who were objectively isolated from both family and friends (*B* = 0.12, *SE* = 0.18, *t* = 0.67, *p* = 0.51) and women who were objectively isolated from family only (*B* = −0.10, *SE* = 0.11, *t* = −0.93, *p* = 0.36) did not differ from women who were not objectively isolated from either group in their likelihood of being diagnosed with hypertension. Therefore, while there was no significant interaction between objective isolation from family × females on average, simple slope analyses suggest the possible presence of gender differences in hypertension between objective isolation from family only and no objective isolation from family/friends.

## 4. Discussion

Our study is among the first to investigate gender differences in the association between objective and subjective social isolation and hypertension among a nationally representative sample of older adults. Overall, our study found that older adults (both men and women) who were objectively isolated from both family and friends and friends only were less likely to report hypertension than older adults who were not objectively isolated from either group. A significant interaction indicated that the association between objective isolation and hypertension varied by gender.

Prior research has connected social integration to the facilitation of positive health behaviors and decreased mortality [11,42]. This is consistent with recent findings from the National Academies of Science report, “Social Isolation and Loneliness in Older Adults”, which posits that social isolation and loneliness affect the quantity and type of health care services used by older adults, specifically preventative health services [7]. Health maintenance behaviors such as regular doctor’s visits are protective for individuals who are at risk for hypertension or who have already been diagnosed with the condition and are receiving treatment [43]. Those who are socially isolated and less socially integrated may lack interaction with people who would otherwise encourage and remind them to engage in health maintenance behaviors such as engaging in preventative care [43]. Therefore, individuals who are socially isolated are less likely to engage in these preventative health behaviors, which could lead to hypertension being underdiagnosed. Increased social integration would place individuals in contact with others who would encourage health maintenance behaviors.

The data indicated that when subjective isolation was controlled, the association between objective isolation from both family and friends and hypertension was no longer statistically significant. Only the association between objective isolation from family and hypertension maintained statistical significance. This indicates that the effects of objective isolation from family on hypertension are independent of the effects of the perceptions of isolation (subjective isolation). This finding, in combination with the subjective isolation null finding, underscores the importance of social contact and interactions, especially with extended family members, in hypertension risk among older adults. Further, this suggests that the perception of social disconnectedness (subjective isolation) may not be as important as the lack of social interactions (objective isolation) in the context of hypertension risk. Social interactions among family members may play a greater role in hypertension diagnoses, as these interactions are opportunities for family members to remind and encourage older adults to maintain regular doctor’s visits and medical check-ups. Furthermore, family members may have a greater impact on the health of older adults in comparison to friends through social influence and social regulation. These findings are consistent with previous studies that have linked objective isolation to negative health behaviors [13,44,45]. In contrast, the perception of social isolation (i.e., subjective isolation) may be more indirectly related to hypertension, which would explain the null finding for subjective isolation, which was statistically modeled with objective isolation.

Additionally, this significant interaction association between gender and objective isolation on hypertension may be explained within the context of relationship quality. At first glance, this significant interaction effect suggests that gender significantly impacted objective isolation; however, when simple slopes analyses were conducted, more nuanced results were uncovered. The simple effects analysis indicated that men who were objectively isolated from family and friends were less likely to be diagnosed with hypertension compared to men who were not objectively isolated from either group. Interestingly, we also found that men who were objectively isolated from family only had lower odds of a hypertension diagnosis compared to men who were not objectively isolated from either family or friends.

The lower likelihood of hypertension seen in this sample may indicate that men with limited social networks are not engaging in health activities that could reveal a diagnosis of hypertension. For example, men may not be reporting a diagnosis of hypertension because they are not going to a primary care physician and being tested for hypertension, which could explain the lower likelihood of diagnosis. We may be seeing that socially isolated men may be underdiagnosed with hypertension, as prior research has indicated that social networks are key for the maintenance of health behaviors such as primary care follow-up and medical adherence [46,47]. Prior literature supports the idea that social networks and families greatly influence our health behaviors [48]. Specifically, as it relates to hypertension control, social networks have been identified as facilitators of positive self-care activities [49]. Additionally, Cornwell and Waite [50] found that the risks of undiagnosed and uncontrolled hypertension are lower among those with larger social networks who discuss health issues with their network members. This evidence points to the importance of social relationships for men and positive social network support for hypertension diagnosis and control.

In contrast, if we assume older men who are isolated from family only and isolated from both family and friends have lower rates of hypertension in comparison to socially integrated older men while also having equivalent rates of seeing a physician for regular check-ups, there may be greater negative social interactions involved which explains this finding. Negative social interactions are categorized as conflicts, criticisms, and excessive demands that represent social strain in relationships and have a deleterious impact on mental and physical health [34,35]. Older men who are socially isolated may do this to shield/protect themselves from negative interactions. This is not to say that social isolation is in any way protective, but instead, fewer social demands may be associated with more time and energy to be directed towards self-care, which may include positive health behaviors that protect against diseases such as hypertension. This could point to the fact that the isolation and loneliness epidemic has been particularly impactful for a long period of time among men, and thus, they have adjusted their behaviors over time to account for limited social support systems. This result among men demands more attention to the social networks of older men and how they may perceive their social relationships and to understand the social dynamics undergirding these connections. Preceding research by Kemp and colleagues [45] discussed the need to better understand the structures (i.e., social integration and social network structure) and processes (i.e., social support and relational demands) that potentially influence health and well-being.

Lastly, the simple effects analysis indicated that there was no difference in hypertension by isolation status among women. This suggests that social relationships may function differently for older women versus older men. Potentially, this means that older women, regardless of their isolation status, may continuously engage in health-promotive behaviors (including going to the doctor’s office for yearly check-ups) compared to men. More research is warranted on the nature of social relationships among older women, health maintenance behaviors, and hypertension.

This study makes several contributions to the literature on social isolation and health. Few studies on social isolation have focused on hypertension, which is an indicator of future adverse health outcomes [3,28]. Older adulthood is emerging as a critical time to investigate social isolation as individuals who enter this life stage are likely to experience changes in their social networks and potentially more social isolation. Social isolation was conceptualized in this study as objective and subjective, providing a more complete picture of how each type of social isolation contributed to the likelihood of hypertension among this sample. Another strength of this study is the examination of the interaction between social isolation and gender, as it has provided a more nuanced understanding of the differences between social relationships in older men and women as it relates to the likelihood of having hypertension.

Several limitations should be noted in the study. All measures in the study were self-reported, which are susceptible to recall and social desirability biases. Specifically, using more clinically based measures of hypertension may capture nuances in the relationship between this condition and social isolation. As this was a secondary data analysis, researchers are unsure if other factors may have impacted self-reported diagnoses of hypertension (i.e., use of anti-depressant medications). Additionally, because the NSAL sampled community-dwelling older adults, study findings may not be generalizable to older adults who are homeless or institutionalized and who tend to have a higher prevalence of social isolation from family and friends. Lastly, no causal inferences can be drawn concerning the association between social isolation and hypertension, given the cross-sectional data. Future research should use longitudinal data to determine the relationship between social isolation and hypertension over time. We compared the sociodemographic characteristics of respondents who were included in the analyses against the characteristics of respondents who were dropped from the analyses due to missing data. We found that respondents who were included in the analyses were younger and had higher socioeconomic status than respondents who were dropped from the analyses. Thus, our findings may have limited generalizability to groups excluded from the analytic sample.

## 5. Conclusions

Objective isolation had an unexpected association with the likelihood of hypertension, further illustrating the complexities of social isolation and high blood pressure; therefore, more research on this topic among older adults is necessary. Parsing out the inverse relationship between objective/subjective social isolation and hypertension and how these may differ based on context warrants consideration in future research.

In conclusion, this study provides a detailed examination of differences in the association between objective and subjective social isolation and hypertension. Our study demonstrates that objective social isolation from family and friends may be associated with a lower likelihood of a diagnosis of hypertension. Furthermore, we found gender differences in the relationship between objective social isolation and hypertension diagnosis, which indicates that gender may be an important factor in how social isolation is linked to hypertension. Overall, this study reinforces the importance of investigating social isolation and understanding its role in health among older adults in the U.S.

## Figures and Tables

**Table 1:** Demographic characteristics of the sample and distribution of study variables.

	N (%)	Mean (S.D.)	Range
Age		66.72 (8.58)	55–94
Gender			
Male	543 (44.58)		
Female	896 (55.42)		
Race/Ethnicity			
African American	837 (40.73)		
Black Caribbean	304 (2.74)		
Non-Hispanic White	298 (56.53)		
Education		11.69 (3.39)	0–17
Family Income		30,942 (36,493)	0–640,000
Marital Status			
Separated/Divorced/Widowed/Never Married	929 (53.98)		
Married/Cohabiting	494 (46.02)		
Region			
Non-South	619 (43.98)		
South	820 (56.02)		
Physical Activity		2.62 (0.85)	1–4
BMI		28.27 (5.65)	15.00–62.76
Objective Isolation			
Objectively isolated from family and friends	59 (4.65)		
Objectively isolated from family	89 (7.36)		
Objectively isolated from friends	142 (11.03)		
Not objectively isolated from either group	1090 (76.96)		
Subjective Isolation			
Subjectively isolated from family and friends	25 (1.12)		
Subjectively isolated from family	42 (3.15)		
Subjectively isolated from friends	128 (9.9)		
Not subjectively isolated from either group	1182 (85.83)		
Hypertension			
Yes	828 (58.79)		
No	541 (41.21)		
12-Month Major Depressive Disorder			
Yes	49 (4.14)		
No	1319 (95.86)		

Percents and N are presented for categorical variables and Means and Standard Deviations are presented for continuous variables. Percentages are weighted and frequencies are un-weighted.

**Table 2:** Multivariable weighted logistic regression for hypertension among older adults (n = 1278).

	Model 1	Model 2	Model 3	Model 4
	OR (95% CI)	OR (95% CI)	OR (95% CI)	OR (95% CI)
Objective isolation*gender				
Not objectively isolated from either group*gender ^a^	--	--	--	--
Objectively isolated from family & friends*gender	--	--	12.55 (1.97–79.89) **	--
Objectively isolated from family*gender	--	--	1.84 (0.58–5.84)	--
Objectively isolated from friends*gender	--	--	2.01 (0.36–11.18)	--
Subjective isolation*gender				
Not subjectively isolated from either group*gender ^a^	--	--	--	--
Subjectively isolated from family & friends*gender	--	--	--	0.14 (0.01–1.41)
Subjectively isolated from family*gender	--	--	--	0.81 (0.12–5.24)
Subjectively isolated from friends*gender	--	--	--	2.08 (0.45–9.67)
Objective isolation				
Not objectively isolated from either group ^a^	--	--	--	--
Objectively isolated from family & friends	0.20 (0.06–0.65) *	0.29 (0.08–1.07)	0.14 (0.04–0.48) **	0.31 (0.09–1.08)
Objectively isolated from family	0.45 (0.24–0.84) *	0.43 (0.20–0.91) *	0.34 (0.15–0.77) *	0.42 (0.20–0.89) *
Objectively isolated from friends	0.60 (0.27–1.34)	0.71 (0.32–1.57)	0.49 (0.13–1.84)	0.70 (0.31–1.57)
Subjective isolation				
Not subjectively isolated from either group ^a^	--	--	--	--
Subjectively isolated from family & friends	--	0.77 (0.25–2.36)	0.55 (0.16–1.86)	10.40 (.39–74.17)
Subjectively isolated from family	--	1.03 (0.38–2.81)	0.94 (0.34–2.58)	1.44 (0.06–35.70)
Subjectively isolated from friends	--	0.54 (0.27–1.07)	0.53 (0.26–1.07)	0.19 (0.01–2.71)
Gender				
Men ^a^	--	--	--	--
Women	0.93 (0.52–1.64)	0.89 (0.49–1.61)	0.73 (0.38–1.39)	0.86 (0.44–1.66)
Age	1.03 (0.10–1.06)	1.03 (0.10–1.06)	1.03 (0.10–1.06)	1.03 (0.10–1.06)
Race/Ethnicity				
African American ^a^	--	--	--	--
Black Caribbean	0.92 (0.54–1.77)	0.92 (0.52–1.63)	0.91 (0.50–1.66)	0.91 (0.51–1.61)
Non-Hispanic White	0.66 (0.45–0.96) *	0.65 (0.45–0.96) *	0.64 (0.44–0.95) *	0.66 (0.45–0.96) *
Education	0.93 (0.87–1.00)	0.93 (0.87–1.00)	0.93 (0.87–0.10) *	0.93 (0.87–1.00)
Family income	1.02 (0.76–1.35)	1.01 (0.76–1.34)	0.98 (0.75–1.28)	1.01 (0.77–1.34)
Marital status				
Separated/divorced/widowed/never married ^a^	--	--	--	--
Married/cohabiting	1.21 (0.84–1.73)	1.19 (0.80–1.75)	1.24 (0.85–1.81)	1.21 (0.81–1.80)
Region				
Non-South ^a^	--	--	--	--
South	1.44 (0.92–2.26)	1.44 (0.91–2.29)	1.46 (0.92–2.31)	1.41 (0.90–2.21)
BMI	1.07 (1.01–1.13) *	1.07 (1.01–1.13) *	1.07 (1.01–1.13) *	1.07 (1.01–1.13) *
Depression	2.38 (1.00–5.61) *	2.33 (0.99–5.50)	2.20 (0.91–5.33)	2.27 (0.90–5.71)
Physical activity	0.81 (0.67–0.98) *	0.79 (0.65–0.95) *	0.78 (0.64–0.94) **	0.79 (0.64–0.96) *

*Note.* OR = odds ratio, 95% CI = 95% confidence interval. ^a^ Reference Category. * *p* < 0.05; ** *p* < 0.01.

## Data Availability

The dataset is available from the Inter-University Consortium for Political and Social Research’s website (https://doi.org/10.3886/ICPSR20240.v8) (accessed on 12 February 2025).
